# Towards standardised cross-sectoral agreements for data depositing and data access in Trusted Research Environments: streamlined contracting to advance public benefit research.

**DOI:** 10.23889/ijpds.v9i5.2627

**Published:** 2024-09-18

**Authors:** Brian Rowe, Esther Yang, Shandra Doran, Michelle Graham, Sean van Diepen, Josh Raizman, Albert Tsui

**Affiliations:** 1 University of Alberta; 2 Alberta Health Services; 3 Alberta Precision Laboratories

## Introduction

This study evaluated the operational and clinical outcomes for patients presenting to an emergency department (ED) with chest pain using a 2-hour high-sensitivity troponin T (hs-TnT) protocol compared to an existing 3-hour hs-TnI protocol.

## Methods

This retrospective cohort study used eight population-based linked health administrative datasets for adult patients presenting to a Canadian urban ED with chest pain over a two-year period. The primary outcome was ED length of stay (LOS). Secondary outcomes included health outcomes within 30 days.

## Results

Overall, 5426 patients were included; 2879 and 2547 in the hs-TnI group and hs-TnT groups, respectively. The cohorts were similar: the median age was 58 years, 55% male, and comorbidities matched. Delays in physician initial assessment (PIA; +54 min; 95% CI: 42.2-65.8) and patients leaving without being seen increased (7% vs 12.5%; P < 0.0001) due to worsening overcrowding. The median ED LOS increased from 448 to 481 minutes after hs-TnT implementation (median difference = 33.0; 95% CI: 19.9 to 46.1), although time to second tests decreased by 54 minutes. At 30 days, there were no differences in readmission or death before and after the intervention.

## Conclusion

Implementation of a 2-hour hs-TnT protocol resulted in mixed outcomes: increased PIA and ED LOS, yet faster time to second test. The journey through the ED for patients with chest pain is complex and affected by issues beyond the assay type and the timing of repeat Tn measurements. ED overcrowding needs to be addressed for efficiency to be realized in this setting.

